# Pathophysiology of Chronic Liver Disease Development

**DOI:** 10.3390/ijms23063385

**Published:** 2022-03-21

**Authors:** Malin Fromme, Pavel Strnad

**Affiliations:** Medical Clinic III, Gastroenterology, Metabolic Diseases and Intensive Care, University Hospital RWTH Aachen, Pauwelsstr. 30, 52074 Aachen, Germany; mfromme@ukaachen.de

Chronic liver disease is a major public threat and the second leading cause of loss of working life years in Europe [1]. It is often clinically unapparent and affects the most vulnerable parts of our society [1]. Moreover, the leading causes of liver mortality are rapidly changing due to achievements in the treatment of viral hepatitis, the epidemic of obesity, and the widespread consumption of alcohol. To shed more light on this silent, life-threatening process, we created this Special Issue focusing on factors underlying liver disease development and progression.

Alcoholic liver disease (ALD) is the leading cause of liver death and the responsible mechanisms were reviewed by Liu et al. [2]. The manuscript nicely illustrates the complex interplay between genetic, epigenetic, and environmental factors that are at the center of every chronic liver disease. It also highlights several key events such as the importance of oxidative stress, the gut–liver axis, or the regenerative response [2].

This Special Issue pays close attention to the role and potential of microRNAs (miRNAs), short non-coding RNAs involved in intra- and extracellular signaling pathways and post-transcriptional regulation of gene expression [3]. MiRNAs do not only contribute to injury-related remodeling of the liver termed as fibrosis/fibrogenesis, but they also constitute attractive markers of disease stage and activity. In their review paper, Tadokoro et al. summarized the effects of miRNAs both promoting and inhibiting liver fibrogenesis, as well as their importance in end-stage liver disease characterized by liver cirrhosis and its related complications [3]. They also emphasized the potential of miRNAs as targets of anti-fibrosis therapy [3]. Their work is nicely complemented by a review from Mohr et al., who elaborated on the importance of different miRNAs in hepatocarcinogenesis [4]. Hepatocellular carcinoma (HCC) typically develops in individuals with cirrhosis and advanced liver fibrosis and is responsible for ~20% of liver-related deaths [1]. It arises due to a long-term liver injury that results in an increased amount of DNA damage and ultimately in a malignant transformation. In HCC, miRNAs affect the transition from liver cirrhosis to cancer and because of that, constitute a potential therapeutic leverage. At the same time, they might be useful for patient stratification [4].

In addition to both review articles, Yang et al. directly demonstrated the usefulness of a miRNA induction in mice fed with a methionine-choline-deficient diet (MCD) that is used as a model of fatty liver disease. This is particularly relevant since non-alcoholic fatty liver disease (NAFLD) represents not only a leading, but also a rapidly growing cause of chronic liver disease and is sometimes referred to as the “first pandemic” of the 21st century [5]. Compared to wild-type mice, MCD-fed animals with miRNA-29a overexpression displayed reduced signs of liver injury, steatosis, and fibrosis. As an underlying mechanism, miR-29a inhibited glycogen synthase kinase 3 beta (*Gsk3b*) and repressed sirtuin 1-mediated mitochondrial biogenesis. These events were associated with alleviated mitochondrial proteostatic stress [6]. While these data are encouraging, confirmation in an independent animal model is needed since MCD does not fully resemble the features of human NAFLD.

In another attempt to characterize the factors responsible for NAFLD development, Hohenester et al. turned to the “American lifestyle-induced obesity syndrome” (ALiOS) diet mouse model and examined the contribution of the NLRP3 inflammasome and its effectors IL-1 and IL-18. NLRP3 is a so-called pattern recognition receptor that detects danger signals released from damaged cells/bacteria and activates downstream cytokines such as IL-1 and IL-18 [7,8]. Hohenester et al. demonstrated that the changes in lipid profile seen in the ALiOS model reflected the alterations seen in human NAFLD, and that the loss of IL-18 but not IL-1 protected from early liver damage. Therefore, NLRP3 activation and IL-18R-dependent signaling represent potential modulators of liver injury in NAFLD [8].

An experimental paper from Lunova et al. assessed the functional relevance of two different interferon lambda genes, IFNL3 and IFNL4 [9]. Interferons are key drivers of antiviral immunity and consequently, their genetic variants were shown to mediate host response to hepatitis infection [10]. The downstream effects of IFNL3/IFNL4 in transfected cell lines were similar and seemed to be dependent on the established interferon receptors IL10R2 and IFNLR1 [9]. Altogether, these data did not support the previous hypothesis of IFNL4-specific non-canonical signaling and demonstrated that functional studies conducted with tagged proteins should be interpreted with caution [9].

Last but not least, Rabekova et al. took advantage of a large cohort of patients with liver cirrhosis and studied whether genetic variants in the alpha1-antitrypsin gene affect the susceptibility to development of HCC [11]. In line with previous studies, the heterozygous Pi*MZ genotype was overrepresented in the cirrhotic subjects compared to controls, while the occurrence of the genotype Pi*MS did not differ significantly [11,12]. In contrast, both genotypes were less common in cirrhotics with HCC compared to the ones without [11]. While an independent confirmation is needed, these data might be useful for clinical management and counseling of these individuals.

Altogether, this Special Issue accurately illustrates the current developments in Hepatology such as a focus on personalized medicine with individualized prediction of a disease course as well as the need for stratification of patients into different risk groups. It also reflects the importance of miRNAs, the complexity of inflammatory responses, as well as the growing attention to alcoholic/non-alcoholic liver disease as the leading causes of liver-related mortality.

## Abbreviations

AATD	Alpha-1 antitrypsin deficiency
ALD	Alcoholic liver disease
Gsk3b	Glycogen synthase kinase 3 beta
HCC	Hepatocellular carcinoma
HCV	Hepatitis C virus
IFNL3	Interferons lambda 3
IFNL4	Interferons lambda 4
IL	Interleukin
ISG	Interferone stimulated genes
JAK	Tyrosine-protein kinase
MS	Heterozygous AAT genotype with mutant SERPINA1 allele variant termed ‘S’
MZ	Heterozygous AAT genotype with mutant SERPINA1 allele variant termed ‘Z’
NAFLD	Non-alcoholic fatty liver disease
NASH	Non-alcoholic steatohepatitis
NLRP3	NLR family pyrin domain containing 3
SIRT1	Sirtuin 1
STAT	Signal transducer and activator of transcription
UTR	Untranslated region
UPR	Unfolded protein response
